# Synthesis, Characterization and Photocatalytic Performance of Calcined ZnCr-Layered Double Hydroxides

**DOI:** 10.3390/nano11113051

**Published:** 2021-11-13

**Authors:** Somia Djelloul Bencherif, Juan Jesús Gallardo, Iván Carrillo-Berdugo, Abdellah Bahmani, Javier Navas

**Affiliations:** 1 Laboratory of Chemistry of Inorganic Materials and Applications (LCMIA), University of Science and Technology of Oran-Mohamed Boudiaf USTO MB, BP 1505, El Mnouer 31035, Algeria; somia.djelloulbencherif@univ-usto.dz (S.D.B.); abahmani2002@yahoo.fr (A.B.); 2 Departamento de Química Física, Facultad de Ciencias, Universidad de Cádiz, Puerto Real, E-11510 Cádiz, Spain; jj.gallardo@uca.es (J.J.G.); ivan.carrillo@uca.es (I.C.-B.)

**Keywords:** layered double hydroxides, 2D materials, metal oxides, photocatalysis

## Abstract

The development of new materials for performing photocatalytic processes to remove contaminants is an interesting and important research line due to the ever-increasing number of contaminants on our planet. In this sense, we developed a layered double hydroxide material based on Zn and Cr, which was transformed into the corresponding oxide by heat treatment at 500 °C. Both materials were widely characterized for their elemental composition, and structural, morphological, optical and textural properties using several experimental techniques such as x-ray diffraction, x-ray photoelectron spectroscopy, scanning and transmission electron microscopy, Fourier transform infrared spectroscopy, UV-vis spectroscopy and physisorption techniques. In addition, the photocatalytic activity of both materials was analysed. The calcined one showed interesting photocatalytic activity in photodegradation tests using crystal violet dye. The operational parameters for the photocatalytic process using the calcined material were optimised, considering the pH, the initial concentration of the dye, the catalyst load, and the regeneration of the catalyst. The catalyst showed good photocatalytic activity, reaching a degradation of 100% in the optimised conditions and showing good performance after five photodegradation cycles.

## 1. Introduction

Currently, aquatic ecosystems, human health and the environment are being threatened by coloured contaminants produced in industries such as printing, food production, cosmetics, laundry a particularly the industry textile. In these applications, dyes are widely used due to their chemical stability, ease of synthesis and variety. However, these dyes are one of the main contributors to pollution when released into the environment [1,2,3]. The release of these compounds into industrial wastewater increases their bio-toxic and carcinogenic effects on humans upon contact with the body. The cornea and conjunctiva can be permanently damaged if the eyes come into direct contact with them [4]. Specifically, crystal violet (CV) dye is a triphenylmethane cationic dye [5] used for direct staining and printing in the textile industry, so it is commonly found in the wastewater produced. In addition, it is a toxic, genotoxic and carcinogenic pigment that causes various types of cancer and eye irritations [6,7,8]. Moreover, CV is one of the brightest classes of dye that is soluble in water and hence, CV concentrations less than 1 mg L^−1^ produce a clear colour that shows an inhibitory effect on photosynthesis [9]. Due to these adverse effects of CV dye on public health and the environment, the treatment and disposal of contaminated water have attracted a great deal of attention from the scientific community, which is devoting all its efforts to solving this problem, using several conventional methods [10] such as advanced oxidation, filtration, coagulation, flocculation, microbial degradation [11], ozonation, photocatalysis and adsorption. Among these methods, heterogeneous photocatalysis is currently one the methods most studied by many researchers in the field of the elimination of organic and inorganic pollutants in wastewater because it is considered to be a green, efficient, economical and promising chemical method. This process has attracted more attention due to its capacity for complete mineralization [12,13] and the light-stimulated degradation of organic and inorganic pollutants [14] (dyes, pesticides, metals, etc.). Heterogeneous photocatalysis uses semiconductor materials as photocatalysts, for example layered double hydroxides, metal oxides semiconductors and nanocomposites.

Thus, one type of material used as a photocatalyst are layered double hydroxides (LDHs), also called hydrotalcite-like compounds, characterized by layered structure of the general formula ${\left[M_{1-x}^{2+}\;N_{x}^{3+}\;{\left(OH\right)}_{2}\right]}^{x+}A_{x/n}^{n-}\cdot mH_{2}O$, where M^2+^ = Zn^2+^, Ni^2+^, Co^2+^, Cu^2+^, Mg^2+^; N^3+^ = Cr^3+^, Fe^3+^, Al^3+^; A^n−^ is an interlayer of exchangeable anions such as SO_4_^2−^, NO_3_^–^, CO_3_^2–^ Cl^–^, I^–^, ClO^–^,… and *m* is a number of water molecules [15]. Several conventional methods such as co-precipitation, hydrothermal, urea hydrolysis and ion exchange can be used for the synthesis of LDH materials [16]. Recently, synthetic layered double hydroxides have proven to be promising photocatalysts due to their non-toxic and environmentally friendly nature, cost-effectiveness, efficiency in performing redox reactions with narrow sensitive band gaps, and the fact that they are easy to regenerate [17]. In addition, LDH can be calcinated at different temperatures, which converts them into mixed metal oxides by structural destruction, removing the compensating anions from the interlayer space. This calcination process endows LDH materials with a very important property called the ‘memory effect’. This allows LDH materials to be easily regenerated, obtaining again the layered structure, by bringing the calcined product into contact with an anionic solution containing the desired anion to be intercalated. The calcined materials have been shown to be highly active in the photodegradation of organic pollutants [18]. Particularly, according to recent research, calcined LDH can be a support and/or a potential catalyst for photocatalytic reactions [19,20,21] due to several of its properties. For example, its large specific surface area favours the immobilization of catalytically active species on its surface in addition to the adsorption of pollutants [22], and the electron transfer of the metals of calcined LDH [23]. In addition, they show a high anionic retention capacity, a simple thermal regeneration process and high porosity, which has led to more and more interest among the environmental community. In addition, LDH materials based on Zn and Cr have been synthesized and tested for use in photocatalytic applications, using several anions, such as Cl^−^, SO_4_^2−^, and CO_3_^2−^ [24], and also being modified by including Ag and Ag_3_VO_4_ nanoparticles which led to synergistic effects improving the photocatalytic performance [25] or Tb nanoparticle for enhancing the photocatalysis process under visible light [26]. Therefore, the possible use of LDH-based materials joined with other materials for generating heterojunction effects can be promising in the photocatalytic process for degradation of contaminants. In this work, we developed new LDH materials based on Zn and Cr and then calcined the material to obtain a mixture of oxides which can generate heterojunction effects and improve the photocatalytic activity.

In the present study, an LDH material based on Zn and Cr (ZnCr-LDH) was synthesized by the co-precipitation method. Next, calcination at 500 °C was performed to obtain ZnCr-calcined-LDH (ZnCr-500) mixed oxides. The materials ZnCr-LDH and ZnCr-500 were characterized using several techniques, such as UV-Vis spectroscopy, Fourier transform infrared spectroscopy (FTIR), electron microscopy, porosimetry, X-ray photoelectron spectroscopy (XPS) and X-ray diffraction (XRD) in order to determine the properties of materials related with photocatalytic processes. Therefore, their photocatalytic activity was also studied. The photocatalytic degradation of CV was tested under solar simulator irradiation. The effects of pH, catalyst mass and the initial concentration of dye on photocatalytic degradation efficiency were studied. The possible photocatalytic mechanism were also discussed, and finally, the photocatalyst regeneration was analysed.

## 2. Materials and Methods

### 2.1. Reagents

All reagents were from commercial sources and used without further purification. The raw chemical materials Zn (NO_3_)_2_·6H_2_O (purity ≥ 99.0%), Cr (NO_3_)_3_·9H_2_O (purity~99.0%), NaOH (purity ≥ 98.0%), HCl (purity ≥ 98.0%), NaNO_3_ (purity ≥ 99.0%), H_2_O_2_ (30.0% in water) and crystal violet 10 B dye (CV, purity ≥ 90.0%) were purchased from Sigma Aldrich^©^ (Saint Louis, MO, USA).

### 2.2. Synthesis of ZnCr-Layered Double Hydroxides and Oxides

ZnCr-LDH was synthesized by the co-precipitation method at room temperature. According to a simple procedure [27], the two precursor salts, Zn (NO_3_)_2_·6H_2_O (0.027 mol) and Cr (NO_3_)_3_·9H_2_O (0.009 mol), with a molar ratio R = Zn^2+^/Cr^3+^ = 3 were added to 50 mL distilled water. The mixture was then added dropwise to a 2 M NaNO_3_ solution under magnetic stirring. The pH was kept constant at 5 by adding appropriate amounts of both 1 M NaOH and 1 M HCl solutions. The resulting suspension was left under stirring for 1 h at room temperature. Then, the resulting product was processed hydrothermally at 120 °C for 24 h before being collected by centrifugation and washed with distilled water several times. Next, it was dried at 60 °C for 24 h. The powder obtained was the ZnCr-LDH material. This sample was calcined for 5 h at 500 °C. The product was named ZnCr-500.

### 2.3. Characterization of the Catalysts

The ZnCr-LDH and ZnCr-500 materials were characterized for their elemental, crystallographic, morphological, textural and optical properties using several instrumental techniques.

Firstly, the X-ray diffraction (XRD) technique was used to identify the hydrotalcite structure of double lamellar hydroxides, while X-ray photoelectron spectroscopy (XPS) was used to determine the surface composition and the oxidation state and chemical state bonding of the elements in the ZnCr-LDH and ZnCr-500 samples. For XRD measurements, a Bruker^®^ (model D8 Advance A25, Billerica, MA, USA) diffractometer emitting Cu Kα (1.540 Å) radiation and a Lynxeye detector (Billerica, MA, USA) were used. The measuring range was 5° to 90° in the 2θ range, with an accuracy of 0.020°. Energy and current of electrons colliding with Cu anode were 40.0 kV and 40.0 mA. In addition, XPS spectra were recorded using a Kratos^®^ (model Axis UltraDLD, Kyoto, Japan) spectrometer with monochromatized Al Kα radiation (1486.6 eV) and a passage energy of 20 eV and given with an accuracy of 0.1 eV. The binding scale was referred to the C 1s signal at 284.8 eV. In addition, FTIR spectra were measured using a Bruker^®^ (Billerica, MA, USA) spectrometer, model Tensor37. The data were recorded from 400 to 4000 cm^−1^ and at a resolution of 2 cm^−1^. Then, to analyse the morphology of the ZnCr-LDH and ZnCr-500 materials, scanning (SEM) and transmission electron microscopy (TEM) were employed. For scanning microscopy, a Nova NanoSEM 450 microscope supplied by FEI^®^ (Waltham, MA, USA) was used. A FEI^®^ (Waltham, MA, USA) Talos F200X microscope was used for TEM. In addition, energy-dispersive X-ray spectroscopy (EDX) was performed in both electron microscopy techniques. A Au monolayer was used for making the samples conductive. Moreover, textural properties were obtained by means of nitrogen sorption measurements. Surface area was calculated from N_2_ adsorption isotherm according to the BET method, and the pore size was calculated by the BJH method. Furthermore, the micropore area, volume and external surface area were determined using the “t-plot” statistical thickness method. Finally, to determine the band gap of ZnCr materials, UV-vis spectra in diffuse reflectance mode were recorded using an equipment assembled in our laboratory. The system consists of a halogen lamp, model DH-2000-BAL, supplied by Ocean Optics© (Amersham, UK), as an illumination source, and a USB2000+ spectrometer supplied by Ocean Optics© (Amersham, UK).

### 2.4. Photocatalytic Activity

Preliminary tests. First, the adsorption-desorption equilibrium was characterized. The mixture (catalyst-dye) was stirred magnetically in the dark for 3 h to achieve this equilibrium. The photolysis test, which analyses the degradation without photocatalyst and under irradiation, was performed for comparison purposes. A total of 60 mL of a crystal violet solution (5 mg/L) was kept under irradiation for 3 h. Then, preliminary tests of the photodegradation of CV dye by photocatalysis were performed by taking 60 mL of the CV solution (5 mg L^−1^) in a Pyrex reactor containing 50 mg of catalyst. Both the photocatalysts, ZnCr-LDH and ZnCr-500, were tested. The systems were exposed to irradiation using a solar simulator (Abet Technologies©, model Sun 3000, Milford, CT, USA) illuminating with 1 sun at room temperature under magnetic stirring. The illumination is above the photocatalysis cell. In this setup, no appreciable amount of catalyst remained at the bottom of the reaction vessel. Moreover, the stirring allows to homogenize the dispersion of the photocatalyst in the CV dye solution, and to homogenize the radiation that arrives to all the system. After irradiation, 3 mL of the solution was taken and centrifuged. The pH during the photocatalysis tests were measured by using a pH-meter (Crison^©^, model PH25, Barcelona, Spain) at each extraction of the aliquot for measuring, but the changes in the pH were slight in all cases. Moreover, after every photocatalysis test, the mass of the photocatalyst was measured after centrifugation to analyse its possible dissolution, but this did not occur in any case.

The adsorption-desorption, photolysis and photocatalytic tests were analysed quantitatively by measuring the absorption band at 591.9 nm using a UV-Vis spectrophotometer, model USB2000+, supplied by OceanOptics^©^ (Amersham, UK). Consequently, tests included the degradation of crystal violet 2B (with an absorption band typically at 587 nm).

Analysis of the pH effect. The effect of the pH on the photocatalysis was analysed. To this end, photocatalytic performance was studied using 50 mg of the catalyst and 60 mL of a 5 mg L^−1^ solution of CV at pH = 3, 4, 5 and 7. The irradiation time was 3 h and the equipment described above was used. The most appropriate pH value was defined in these tests. The variation on the pH did not generate the dissolution of the photocatalyst material in the medium.

Effect of the initial concentration of the CV solution. The photocatalytic performance using 50 mg of the catalyst, at pH = 7, and 60 mL of solutions of CV with a concentration of 5, 10, 15 and 30 mg L^−1^ was analysed. The irradiation conditions were the same as in the previous test.

Effect of the amount of catalyst. The photocatalytic performance using 60 mL of a 5 mg L^−1^ solution of CV, and 20, 40, 50 and 70 mg of the catalyst, at pH = 7 was analysed. The appropriate amount of the photocatalyst was determined. The irradiation conditions were the same as in the previous tests.

Regeneration test. Five degradation cycles were performed to evaluate the stability of the catalyst and the efficiency of the degradation process analysed. The tests were performed using 50 mg of the catalyst, a CV dye concentration of 5 mg L^−1^, a pH = 4, and an irradiation time of 180 min. After each cycle, the catalyst was washed with ionised water.

## 3. Results and Discussions

### 3.1. Material Characterization

**Figure 1:**
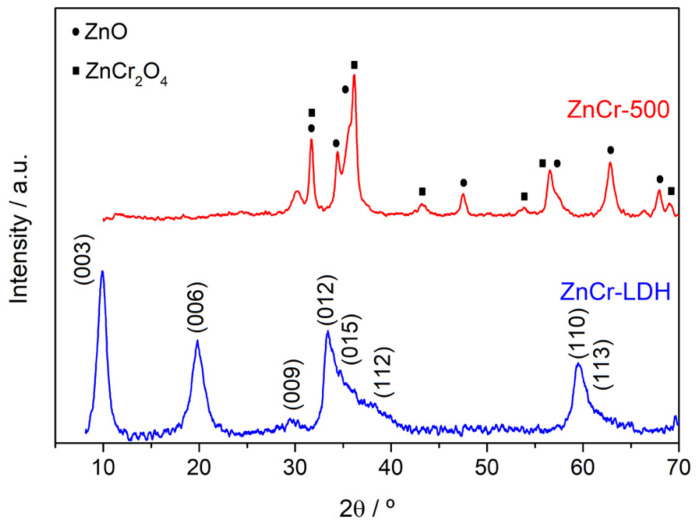
X-ray diffraction patterns for ZnCr-LDH and ZnCr-500 samples.

The XRD patterns of the synthesized ZnCr-LDHs samples and the product derived from its calcination, ZnCr-500, are shown in Figure 1. All the diffraction peaks corresponding to ZnCr-LDH can be indexed on a hexagonal structure with an R3m space group, revealing that the LDHs are crystallized in a rhombohedral structure. This diffractogram shows sharp and symmetric reflections for (003), (006) and wide and asymmetric reflections for (009), (012), (015), (112), (110) and (113) planes, which are characteristic of hydrotalcite-like compounds [28]. The prepared hydrotalcite nanosheets present good crystallinity and high purity.

For this type of material, the interlayer space corresponding to d003 is evaluated at 8.857 Ǻ, which can be assigned to the intercalation of nitrate ions and the extent of hydration between the inorganic lamellae. The cell parameters are commonly evaluated so that c = 3d (003) = 26.571 Å and a = 2d (110) = 3.094 Å. The value a is equivalent to the mean distance between adjacent cation centres and can be correlated with the average radii of metal cations in the layers.

The diffractogram relating to the calcined LDHs (ZnCr-500) shows the disappearance of the diffraction peaks of the initial phase due to the destruction of the lamellar structure by dehydration and denitration during heat treatment at 500 °C. On the other hand, we note the appearance of new peaks characteristic of less crystalline phases. The positions of these peaks fit well with the typical diffraction lines for ZnO (JCPDS Card #36-1451), and ZnCr_2_O_4_ spinel (JCPDS no. 73-1962) [29]. The choose of a ratio R = Zn^2+^/Cr^+3^ = 3 is the reason for obtaining both species. The excess of Zn leads to the formation of ZnO and the spinel, and to avoid Cr_2_O_3_. Ratios higher than 3 leads to the formation of Zn (OH)_2_, which is not an interesting specie for photocatalytic tests [30]. Therefore, we expect that the formation of two phases, ZnO and spinel, leads to heterojunction effects which can improve the photocatalytic performance of the calcined sample.

**Figure 2:**
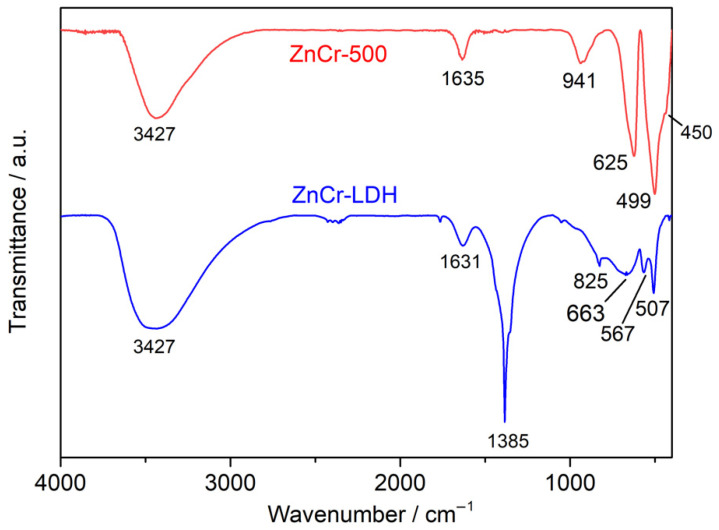
FTIR spectrum of ZnCr-LDH and ZnCr-500 photocatalysts.

The FTIR spectra of the ZnCr-LDH and ZnCr-500 samples are depicted in Figure 2. The wide and intense band at 3427 cm^−1^ is attributed to the stretching vibration of the O-H bond of the hydroxyl groups of layers and interlayer water molecules. The wideness of the band indicates the existence of hydrogen bonds. The weak peak at 1635 cm^−1^ is attributed to the deformation vibration of interlayer water. The strong band observed at 1385 cm^−1^ and another low one at 825 cm^−1^ are assigned to the ν3 and ν2 vibration modes of compensating NO_3_^−^ interlayer anions. In addition, the vibration bands at low frequencies (below 1000 cm^−1^) are attributed to the deformation and translation modes of M-OH and M-O-M bonds forming the sheets of the layered material.

The FT-IR spectrum of the calcined product, ZnCr-500, shows the disappearance of the characteristic bands of nitrates, which confirms the denitration, and of those located at less than 1000 cm^−1^, which are replaced by new characteristic bands of the M-O and M-O-M bonds of the oxides obtained after calcination. The presence and decrease are observed in the intensity of the 3427 cm^−1^ and 1635 cm^−1^ bands, which confirm the presence of water due to the high hydration of the oxides obtained during their storage.

**Figure 3:**
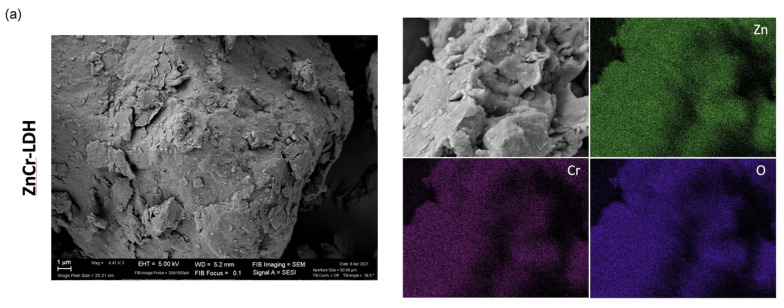

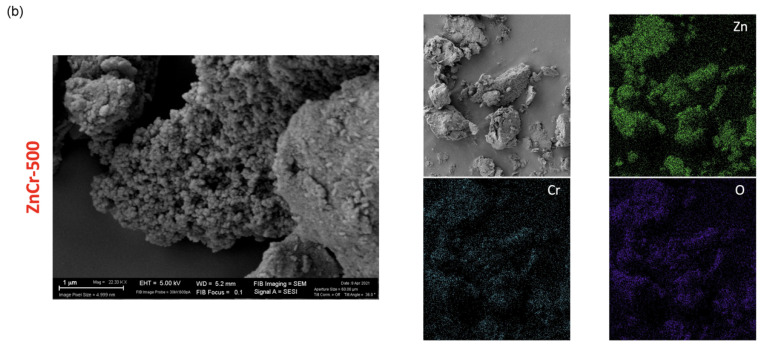
SEM images and EDX mapping of Zn, Cr and O elements for ZnCr-LDH (**a**) and ZnCr-500 (**b**) samples.

Scanning transmission microscopy was performed to analyse the morphology of the samples, and energy-dispersive X-ray spectroscopy to determine their elemental composition. Figure 3 shows SEM images for both samples. The morphology can be seen to change after the calcination. Before the heat treatment, the sample seems to be composed of agglomerated sheets forming big particles (Figure 3a). After calcination, smaller nanoparticles are observed (Figure 3b). This change in the morphology of the samples is better observed from the TEM images below. Finally, the presence of Zn, Cr and O was confirmed for both samples from EDX mapping, as observed in Figure 3.

**Figure 4:**
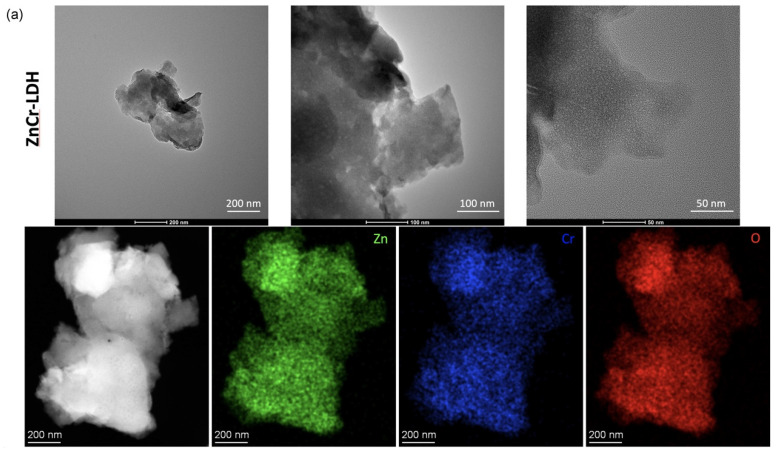

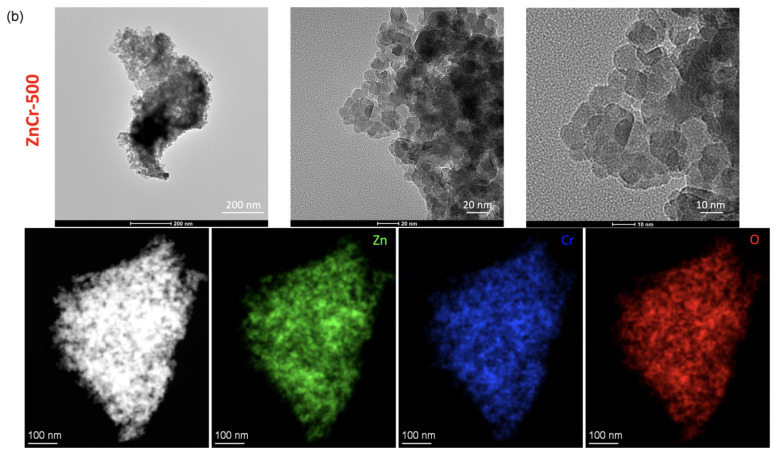
TEM images and EDX mapping of Zn, Cr and O elements for ZnCr-LDH (**a**) and ZnCr-500 (**b**) samples.

Transmission electron microscopy was performed to analyse the morphology of both samples. As is observed in Figure 4a, the non-calcined sample, which is the layered double hydroxide, ZnCr-LDH, presents a layered structure. But as expected, the calcined sample lost this structure, and crystalline nanoparticles of about 10 nm were found (see Figure 4b). In addition, the uniform presence of Zn, Cr and O is observed in both samples, as is shown from the EDX mapping in Figure 4. The presence of O in the ZnCr-LDH sample is due to the hydroxide groups and also to the NO_3_^-^ interlayer anions, as shown in the FTIR spectra above. In the case of the calcined sample, the presence of O is due to the oxide compounds in the sample.

**Figure 5:**
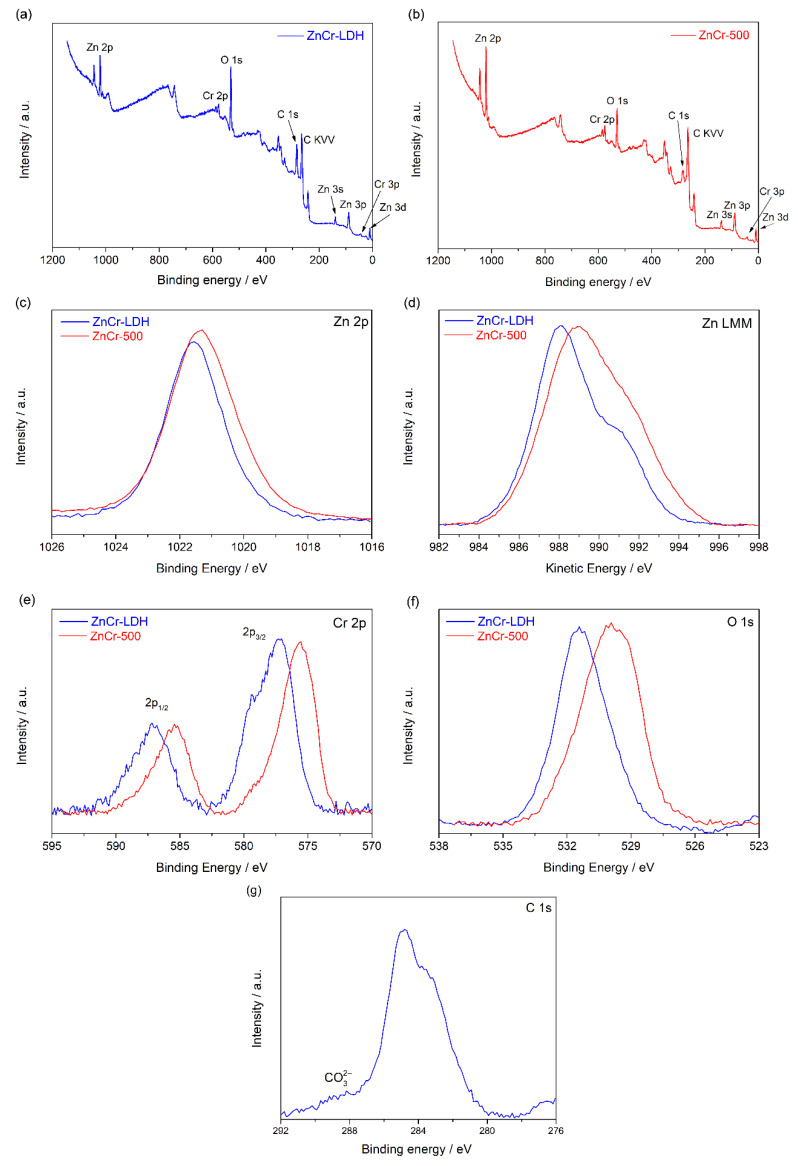
XPS measurements: survey spectra for ZnCr-LDH (**a**) and ZnCr-500 (**b**); Zn 2p region of both samples (**c**); Zn LMM Auger peak (**d**); and Cr 2p (**e**); O 1s (**f**) and C 1s (**g**) regions for both samples, and C 1s for ZnCr-LDH sample.

XPS was used to determine the chemical state bonding for the elements involved in the samples synthesized, that is, the non-calcined LDH and the calcined sample. The presence of Zn, Cr and O is confirmed by analysing the survey spectrum for both samples, as shown in Figure 5a,b. Moreover, Figure 5c shows the XP Zn 2p_3/2_ region for both samples, ZnCr-LDH and ZnCr-500. We can observe a slight shift (about 0.3 eV) towards lower binding energies (BE) for the calcined sample, which is typical for Zn oxides relative to Zn hydroxides [31]. However, the shifts for the Zn 2p signal for different Zn compounds are typically small, so chemical state differentiation can be difficult using only this signal. Thus, the Zn LMM Auger peak was recorded for both samples, and they are shown in Figure 5d. The signal for the non-calcined sample was found at about 987 eV, a typical value for Zn (II) species [31]. A shift of about 1.1 eV towards higher kinetic energy (KE) for the calcined sample is observed, which corresponds to electron states with a lower BE in good concordance with the previous Zn 2p results shown. This confirms that the calcined and non-calcined samples have different chemical states.

Figure 5e shows the Cr 2p spectra for both samples. The Cr 2p_3/2_ contribution for ZnCr-LDH appears at a BE of 577.3 eV, in good agreement with previous results of Cr (III) hydroxide [32]. For the calcined sample, ZnCr-500, the Cr 2p_3/2_ contribution is shifted to a lower BE at 575.6 eV, which is close to that value previously reported for zinc (II) chromium (III) oxide [32]. Moreover, Figure 5f shows the O 1s spectra for both samples. The BE of the O 1s signal for the non-calcined samples appears at about 531.4 eV, while the signal for calcined samples is found at 529.8 eV. This confirms the greater presence of oxides in the calcined sample and hydroxides in the non-calcined one, which is in concordance with the results obtained for the Zn and Cr signals. Finally, Figure 5g shows the C 1s contribution for the ZnCr-LDH sample. Th typical signal for adventiouos C is found, but also, a residual contribution of carbonate species due to the realisation of the synthesis at atmospheric conditions is observed at BE higher than 288 eV.

**Figure 6:**
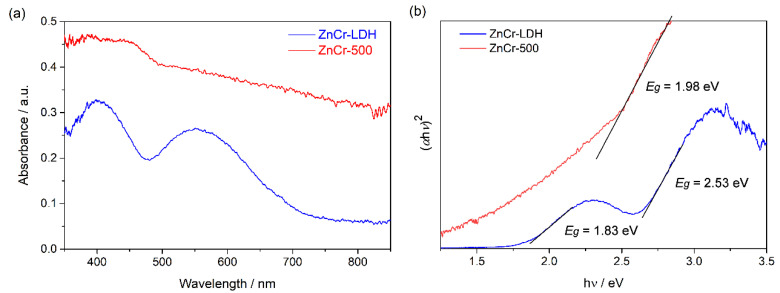
(**a**) UV-Vis absorbance from diffuse reflectance measurements; and (**b**) Tauc plots for ZnCr-LDH and ZnCr-500 photocatalysts.

The optical properties of the synthesized materials were also studied by means of UV-Vis spectroscopy in diffuse reflectance mode. The spectra of ZnCr-LDH and ZnCr-500 samples are shown in Figure 6a. ZnCr-LDH sample shows two absorption bands in the visible region of the spectrum, about 400 nm and 559 nm. In addition, ZnCr-500 shows an absorption band of around 448 nm. Moreover, the absorption for the calcined sample, ZnCr-500, is higher than for the non-calcined sample in all the visible range of the spectrum. The optical bandgap energy of ZnCr-LDH and ZnCr-500 was obtained from the Tauc plot using the equation

(1) $${\left(\alpha h\nu \right)}^{n}=a_{0}\;\left(h\nu -E_{g}\right),$$

where *α* is the absorption coefficient, h*ν* is the photon energy, *a*_0_ a specific constant, and *E_g_* is the bandgap energy. *n* is given by the nature of transition: for a direct gap, *n* = 2, and for an indirect gap, *n* = 1/2 [33]. In this study, *n* = 2 due to the presence of a direct transition. The band gaps were determined as 2.53 eV and 1.83 eV for ZnCr-LDH, and 1.98 eV for ZnCr-500, as is shown in Figure 6b.

**Figure 7:**
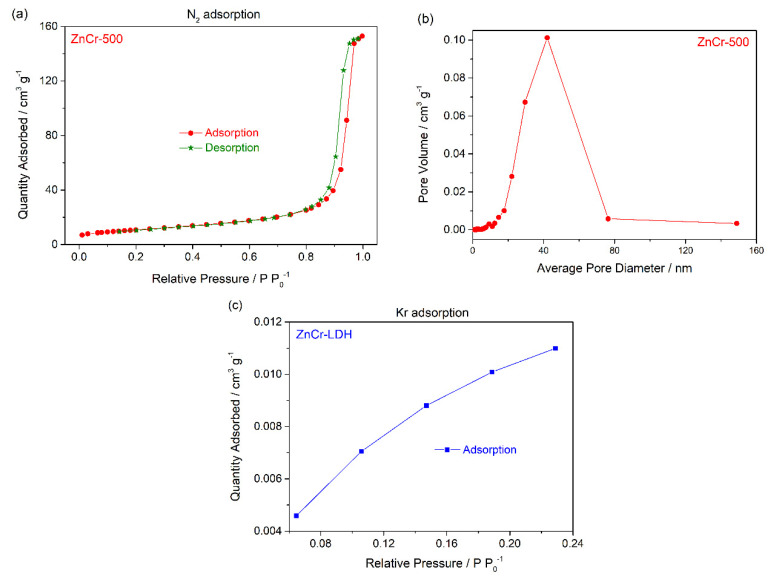
(**a**) N_2_ adsorption-desorption isotherm; and (**b**) pore size distributions obtained for ZnCr-500 sample; (**c**) Kr adsorption isotherm for ZnCr-LDH.

For more details on the textural properties of the material, nitrogen sorption was measured to estimate the pore diameter, specific surface area and pore volume. Figure 7a displays the N_2_ sorption isotherm of ZnCr-500 calcined at 500 °C. The layered sample, ZnCr-LDH, did not adsorb N_2_ in this test because of the small size of the pores. The isotherm for the ZnCr-500 sample exhibits the shape of a type II isotherm, characteristic of mesoporous materials according to the IUPAC classification (International Union of Pure and Applied Chemistry, IUPAC). The shape of the curve can be explained by the existence of pores between the particles of nanometric size, which form an interparticle porosity. This behaviour is characteristic by a type H3 hysteresis loop, as is observed in Figure 7a. The corresponding pore size distribution is shown in Figure 7b. A notable wide distribution centred below 40 nm is observed. In addition, the Kr adsorption test was performed for the ZnCr-LDH sample. The isotherm obtained is shown in Figure 7b. It shows the quantity absorbed is very low in comparison with the ZnCr-500 sample, as discussed previously. Finally, the texture parameters for ZnCr-500 obtained from the N_2_ adsorption test are shown in Table 1. The specific surface area was about 38.45 m^2^ g^−1^, while in the case of the ZnCr-LDH sample, the specific surface area from the Kr adsorption test was 0.06 m^2^ g^−1^, confirming the small surface area of the non-calcined, layered sample. We have to keep in mind that the preparation of the sample previous to the test can modified the surface in the case of the ZnCr-LDH sample, due to the fact that the interlayer spaces are occupied by water molecules and interlayer anions. However, from a qualitative perspective, we can conclude that the calcined sample, ZnCr-500, shows a much higher specific surface area.

### 3.2. Photocatalytic Activity

**Figure 8:**
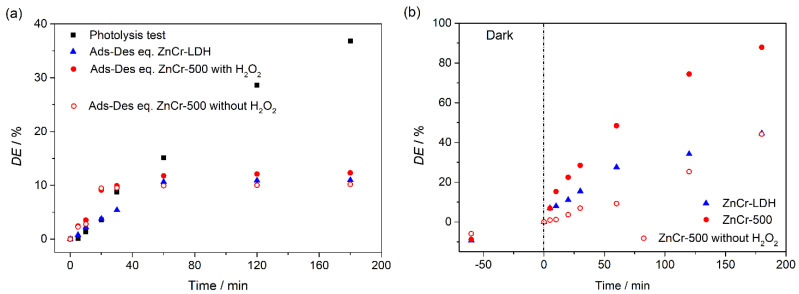
(**a**) Photolysis and adsorption-desorption equilibrium tests, and (**b**) photocatalysis activity tests, for ZnCr-LDH and ZnCr-500 samples.

The photocatalytic degradation of CV dye was analysed by means of UV-vis spectroscopy, recording the absorbance at 591.9 nm as is described previously. From the spectra recorded, degradation efficiency, *DE* (%), was calculated by

(2) $$DE\;\left(\%\right)=\frac{\left(A_{0}-A_{t}\right)}{A_{0}}*100,$$

where *A*_0_ and *A_t_* are the absorbance values before starting the photocatalytic reaction and after time *t*, respectively. Before the photocatalytic tests, the photolysis process and the adsorption-desorption equilibrium for both photocatalysts were measured. Figure 8a shows the photolysis and the adsorption-desorption tests. The photolysis experiment was performed under the sunlight simulator irradiation (1 sun) in the presence of H_2_O_2_ (0.3 mL), which led to a pH = 4. Tests in absence of H_2_O_2_ were performed, being the adsorption-desorption equilibrium similar to that performed with H_2_O_2_, but the photodegradation using ZnCr-500 in the presence of H_2_O_2_ was significantly higher than without H_2_O_2_ (see Figure 8). Therefore, all the tests were performed in the presence of H_2_O_2_. The photolysis process led to a CV degradation of about 36%. In addition, the adsorption-desorption test using both photocatalysts were performed in the dark and in the presence of H_2_O_2_ (0.3 mL, pH = 4). As Figure 8a shows, the adsorption-desorption equilibrium was reached after 60 min for both photocatalysts. The CV depletion from the liquid phase by adsorption was about 12% in both cases. Finally, the photocatalysis tests were performed using both photocatalysts, adding the same amount of H_2_O_2_, and following the procedure described above. The results obtained are shown in Figure 8b. After 180 min of the test, the degradation percentage observed for the non-calcined sample, ZnCr-LDH was slightly higher than in the photolysis test, this sample therefore showing low photocatalytic activity. The ZnCr-500 photocatalyst showed higher activity, reaching a degradation percentage of about 87.8% (see Figure 8b). As is discussed previously, the formation of two phases, ZnO and ZnCr_2_O_4_ spinel, leads to the manifestation of heterojunction effects which can improve the photocatalytic performance. In this case, the heterojunction is an isotype junction because both semiconductors ZnO and ZnCr_2_O_4_ are n-type semiconductor. Therefore, only the ZnCr-500 photocatalyst was used for the photocatalysis tests shown below.

**Figure 9:**
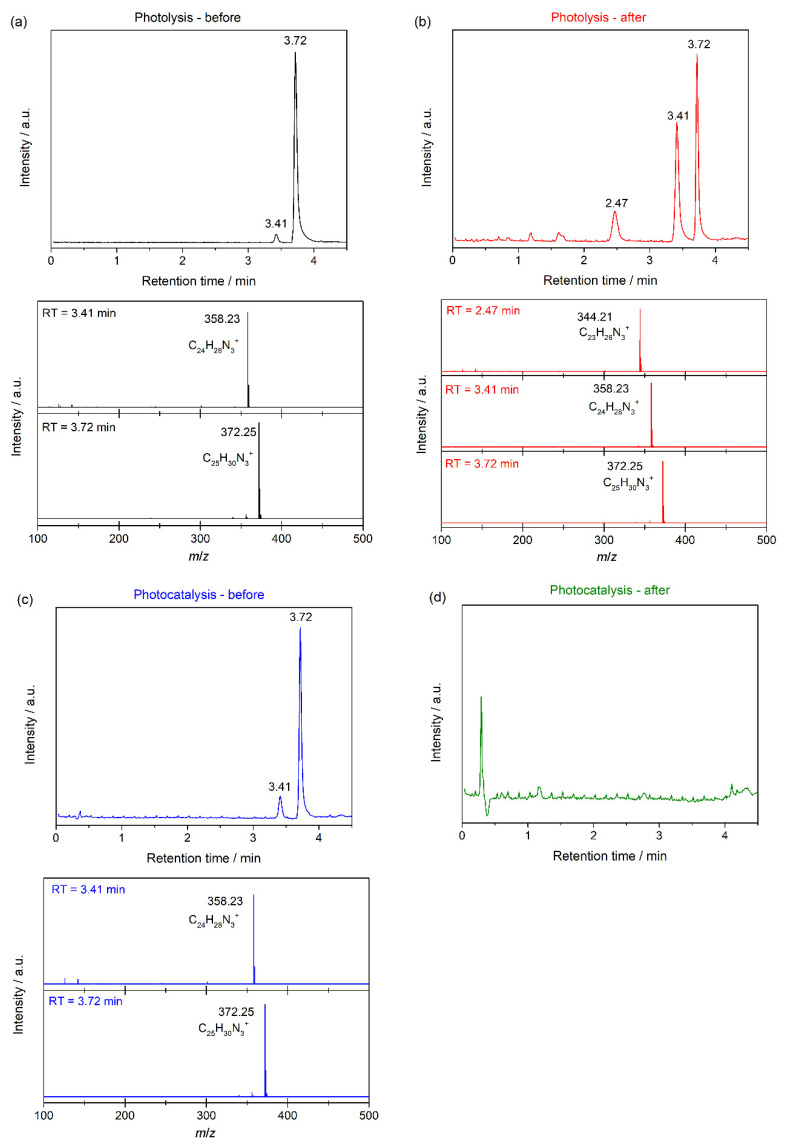
Chromatograms and mass spectra for the solutions (**a**) before and (**b**) after of the photolysis process; and (**c**) before and (**d**) after photocatalysis process.

The products of the degradation for both methods, i.e., photolysis and photocatalysis, were analysed by using HPLC coupled with mass spectroscopy. Figure 9 shows the results of the HPLC-MS measurements performed. In detail, Figure 9a shows the chromatogram for the sample before the photolysis test. Two peaks at retention times of 3.41 and 3.72 min were observed. From the mass spectra for both peaks, we can assign the peak at 3.41 min to *m*/*z* = 358.23, which corresponds to C_24_H_28_N_3_^+^. The peak at 3.72 min, which is the most intense, is assigned to *m*/*z* = 372.25 and to the compound C_25_H_30_N_3_^+^. This is the cation of crystal violet 10 B dye, used in this synthesis. According to the supplier’s data, its purity is about 90%, which is coherent with the presence of the other compound in the chromatogram. This minority compound, C_24_H_28_N_3_^+^, is the cation of the crystal violet 2B. In addition, Figure 9b shows the chromatogram for the solution obtained after the photolysis process. Three peaks are observed, the same peaks for the sample before photolysis, and a new peak at a retention time of 2.47 min. From the mass spectrum, this peak is assigned to C_23_H_26_N_3_^+^, which is the cation of crystal violet 6B. In relative terms, the intensity of the peak at 3.72 min decreased with regard to the peak at 3.41 min, which means that the compound incorporating the C_25_H_30_N_3_^+^ cation is the one that is photodegraded in the photolysis process. In addition, the photocatalysis process was also analysed by HPLC-MS before and after. Figure 9c shows the results for the sample before photocatalysis. As expected, the same peaks are observed as in the test performed before the photolysis test. Figure 9d shows the chromatogram for the sample extracted after the photocatalysis process. As is shown, no peaks are observed in this chromatogram, showing that the degradation of the crystal violet dye was complete in the photocatalysis process, which is coherent with the colourless, transparent solution obtained.

On the other hand, several photocatalysis mechanisms have been reported previously in the literature, including some in which the sensitization of the photocatalyst by the dye occurs [34,35]. The mechanism proposed in this work can be resumed as: (i) the adsorption of CV molecules occurs on the surface of the ZnCr-500 photocatalyst; (ii) which, absorbs photons and the electrons are excited to the conduction band generating electron-hole pairs; (iii) the electrons on the conduction band and the holes in the valence band are responsible for producing reactant species; and (iv) finally, oxidant species oxidize the dye molecules producing the species obtained from HPLC-MS results, which are desorbed from the photocatalyst surface.

In addition, the photocatalytic activity is typically controlled by the band structure of the semiconductors involved in the case of heterojunction formation, in which the energy band coupling can reduce the recombination probability between electrons and holes photogenerated. In this sense, the potential for the conduction (CB) and valence band (VB) for the two semiconductors involved in this work can be calculated as [36]

(3) $$E_{VB}=X-E^{e}+0.5E_{g}$$

(4) $$E_{CB}=E_{VB}-E_{g}$$

where *E_VB_* is the edge potential of the valence band, *E_CB_* is the edge potential of the conduction band, X is the absolute electronegativity of the semiconductor, *E^e^* is the energy of the free electrons in the hydrogen scale (that is ~4.5 eV), and *E_g_* is the band gap energy. X and *E_g_* are 5.71 eV and 1.8 eV for ZnCr_2_O_4_ [37] and 5.76 eV and 3.3 eV for ZnO [38]. Therefore, the VB and CB edges were 2.11 and 0.31 eV for ZnCr_2_O_4_, and 2.91 and −0.39 eV for ZnO. Thus, according to the band edge positions, the holes in the VB of ZnO can be transferred to the VB of ZnCr_2_O_4_. Moreover, the electrons generated in the CB of ZnO can be transferred to ZnCr_2_O_4_. These charge transfers can inhibit the quick recombination of the elentros and holes. These ideas can gel to understand the behaviour of the semiconductor synthesized, but further investigations are needed for a complete understanding of the photocatalityc process.

#### 3.2.1. pH Effect

pH is considered one of the main factors influencing the interactions between the surface of the catalyst and the dye, and therefore, photocatalytic performance is often affected by the pH value. Therefore, the analysis of the effect of pH is essential in photodegradation studies.

**Figure 10:**
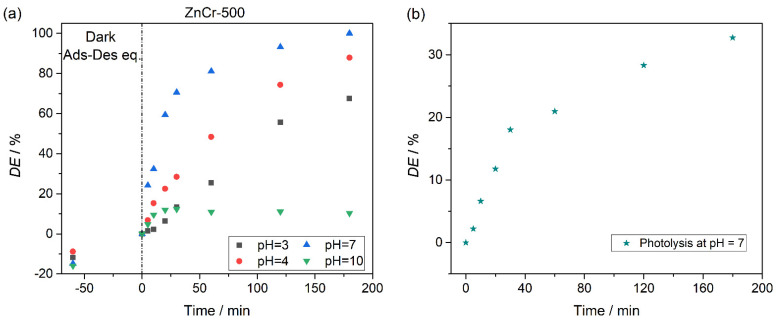
(**a**) Photocatalysis degradation of CV dye according to pH using ZnCr-500 material as photocatalyst. (**b**) Photolysis test at pH = 7.

Photocatalytic experiments were performed in the range of pH 3–10 following the same procedure described above and using the ZnCr-500 sample as the photocatalyst. The corresponding degradation plots are shown in Figure 10. The curves indicate an increase in the rate of degradation from acidic pH to medium (pH = 7). A decrease in degradation efficiency at pH = 10 was observed. In detail, at pH = 3 and pH = 4, degradation percentage of CV dye of about 67.5% and 87.8% was found after 180 min of irradiation. In an acidic medium, the degradation is retarded due to the high concentration of protons [36]. At pH = 10, no photocatalytic degradation was observed. This can be understood by considering that the larger concentration of OH^−^ groups in the solution hinders the movement of the CV anions and its ability to make contact with the catalyst surface, leading to a decrease in the degradation rate [39]. For analysing the photocatalysis activity at pH = 7, photolysis test was performed in order to observe if there is an important contribution of the photolysis. Figure 10b shows the photolysis test at pH = 7, in which the degradation after 180 min was about 33%, similar to that obtained at pH = 4. At pH = 7, the photocatalysis experiment showed good and quick photocatalytic degradation of the CV dye, reaching almost 100% after 180 min of irradiation. Therefore, all the following experiments were performed at pH = 7.

#### 3.2.2. Effect of Initial Concentration of CV Dye

**Figure 11:**
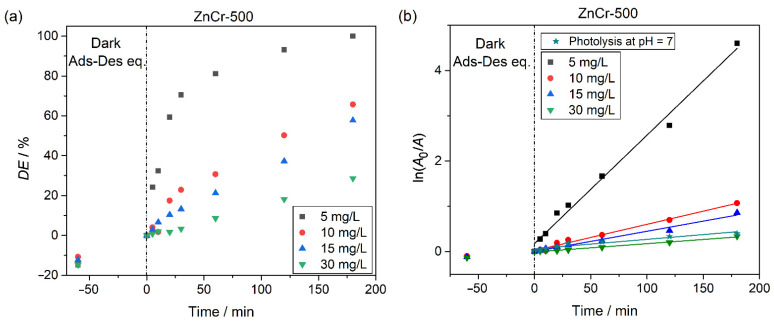
Analysis of the effect of the initial CV dye concentration in the photocatalytic degradation using ZnCr-500 as photocatalyst (50 mg), at pH = 7. (**a**) Plot of the degradation percentage vs. time, and (**b**) kinetics plot for obtaining the rate degradation constant.

The initial concentration of the dye is an important parameter in the performance of wastewater remediation. Thus, the degradation of different CV solutions was studied in the concentration range of 5–30 ppm (5, 10, 15, 30 mg L^−1^) at pH = 7, using 50 mg of ZnCr-500 as the photocatalyst. The results of these degradation tests are shown in Figure 11a. After 180 min of irradiation, the ZnCr-500 catalyst showed the highest degradation efficiency for the lowest concentration of the CV dye, reaching a degradation of about 100%. Figure 11 shows that the higher the CV dye concentration, the lower the degradation percentage, these being 100%, 65.7%, 57.7% and 28.6% for CV concentrations of 5, 10, 15 and 30 mg L^−1^, respectively. The decrease in degradation with higher dye concentrations could be due to the fact that the active catalytic sites were already saturated. Moreover, the rate degradation constant has been estimated considering a pseudo-first order reaction, which kinetics can be described by $ln\left(A_{0}/A\right)=-kKt=k_{app}t$, where *A*_0_ is the initial concentration of CV, *A* is the concentration of CV at the time of irradiation *t*, *k* is the reaction velocity constant, *K* is the adsorption constant of the reactant and *k_app_* is the apparent constant rate [40]. The plot of the $ln\left(A_{0}/A\right)$ vs. time is shown in Figure 11b. The values of the *k_app_* for the tests depending on the CV concentration are shown in Table 2. From these values, the optimal concentration for the degradation of the CV dye of the given catalytic system is 5 mg L^−1^. At this concentration and at pH = 7, the photolysis test shows a rate degradation constant significantly lower than the photocatalytic test, as is observed in Table 2.

#### 3.2.3. Catalyst Mass Effect

**Figure 12:**
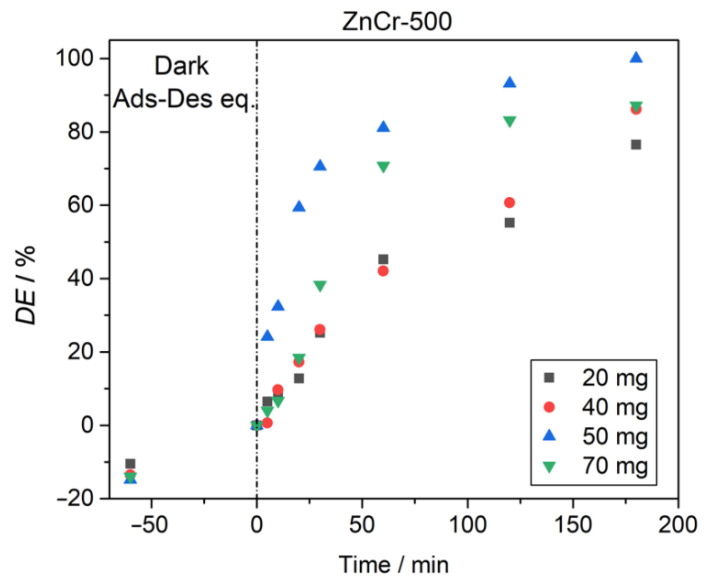
Effect of ZnCr-500 catalyst mass on degradation efficiency of CV (dye concentration 5 mg L^−1^, pH = 7).

A series of experiments were performed to evaluate the optimal catalyst loading by varying the amount of catalyst from 20 to 70 mg using a CV dye concentration of 5 mg L^−1^ at pH = 7. Figure 12 shows the results of these experiments. Using 50 and 70 mg of the ZnCr-500 catalyst, a rapid degradation was observed, reaching a high degradation percentage after 60 min. Meanwhile, using 50 mg of the catalyst, the degradation was practically complete after 180 min. The degradation percentage increased with the photocatalyst mass up to 50 mg. As is observed from the test using 70 mg of photocatalyst, higher masses than 50 mg did not produce any improvement in the degradation. Therefore, 50 mg is the optimal photocatalyst mass for the conditions employed in these experiments.

#### 3.2.4. Regeneration of the ZnCr-500 Catalyst

**Figure 13:**
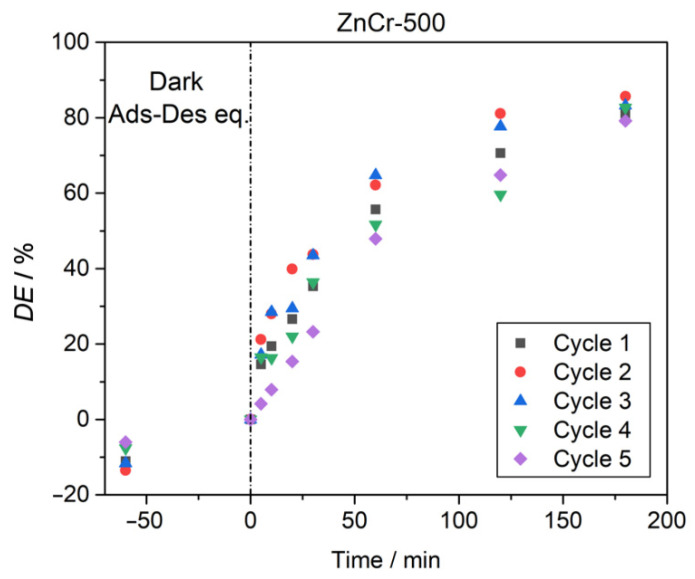
Cyclic degradation of CV dye by using ZnCr-500 catalyst.

For the purpose of the practical implementation of the photocatalyst analysed in this work, it is essential to evaluate its stability and whether it can be reused. Five degradation cycles were performed to evaluate the stability of the catalyst and the efficiency of the degradation process analysed. The tests were performed using 50 mg of the photocatalyst, a CV dye concentration of 5 mg L^−1^, at pH = 4, and an irradiation time of 180 min. The catalyst was washed after each cycle with deionised water. The results are shown in Figure 13. A high stability for the ZnCr-500 photocatalyst can be observed. The decrease in the degradation percentage after four cycles was negligible. A slight decrease was observed after the fifth cycle, but this was probably due to losses of catalyst during the washing process after each cycle.

## 4. Conclusions

In this paper, a Zn and Cr-based layered double hydroxide (ZnCr-LDH) was synthesized and calcined at 500 °C to obtain the corresponding oxide (ZnCr-500). From XRD patterns, ZnCr-LDH presented a hexagonal structure, while in ZnCr-500, the lamellar structure was destroyed by dehydration and denitration during heat treatment at 500 °C. The denitration was confirmed by FTIR. In addition, the morphology was confirmed by electron microscopy. The layered morphology of ZnCr-LDH was observed in TEM images, while the presence of small nanoparticles was obtained after the heat treatment. Moreover, SEM revealed a strong agglomeration in the layered sample. In addition, the homogeneous distribution of Zn and Cr in the samples was confirmed by EDX coupled to electron microscopy techniques, while the expected oxidation states for the cations was confirmed by XPS.

Optical measurements showed band gap energy in the visible region of the electromagnetic spectrum, which means these samples can be useful for photocatalytic processes. The textural analysis by physisorption tests showed that the non-calcined sample had a very low surface area, while the calcined sample, ZnCr-500, showed a high surface area, which is an interesting feature for photocatalysis.

Photocatalytic tests to analyse photodegradation of crystal violet dye showed that the calcined sample is a promising material for the degradation of contaminants, while the layered material showed low photocatalytic activity due to the low surface area. The operational parameters for photocatalytic processes using ZnCr-500 material were optimised considering the pH, the initial concentration of the dye, the catalyst load, and the regeneration of the catalyst. The catalyst showed good photocatalytic activity, reaching a degradation of 100% in the optimised conditions, and it also performed well after five photodegradation cycles.

## Figures and Tables

**Table 1:** Specific surface area, pore size and pore volume for ZnCr-LDH and ZnCr-500 samples.

Sample	Specific Surface Area/m^2^ g^−1^	Average Por Size/nm	Pore Volume/cm^3^ g^−1^
ZnCr-500	38.45 ± 1.00	26.0 ± 3.0	0.232 ± 0.03
ZnCr-LDH	0.06 ± 0.01		

**Table 2:** Rate degradation constant for the tests using different CV dye concentration.

Test	CV Dye Concentration/mg L^−1^	*k_app_*/min^−1^	R^2^
Photocatalyst: ZnCr-500; 50 mg, pH = 7	5	0.0239	0.9881
	10	0.0058	0.9861
	15	0.0045	0.9803
	30	0.0019	0.9880
Photolysis test at pH = 7	5	0.0021	0.9843

## Data Availability

Data available on request due to restrictions e.g., privacy or ethical.
